# Redox Homeostasis in Pancreatic β-Cells: From Development to Failure

**DOI:** 10.3390/antiox10040526

**Published:** 2021-03-27

**Authors:** Štěpánka Benáková, Blanka Holendová, Lydie Plecitá-Hlavatá

**Affiliations:** 1 Department of Mitochondrial Physiology, Institute of Physiology, Czech Academy of Sciences, 142 20 Prague 4, Czech Republic; stepanka.benakova@fgu.cas.cz (Š.B.); blanka.holendova@fgu.cas.cz (B.H.); 2 First Faculty of Medicine, Charles University, Katerinska 1660/32, 121 08 Prague, Czech Republic; 3 Department of Mitochondrial Physiology, Czech Academy of Sciences, Videnska 1083, 142 20 Prague 4, Czech Republic

**Keywords:** redox signaling, oxidative stress, redox homeostasis, pancreatic β-cells, de/differentiation, inflammation

## Abstract

Redox status is a key determinant in the fate of β-cell. These cells are not primarily detoxifying and thus do not possess extensive antioxidant defense machinery. However, they show a wide range of redox regulating proteins, such as peroxiredoxins, thioredoxins or thioredoxin reductases, etc., being functionally compartmentalized within the cells. They keep fragile redox homeostasis and serve as messengers and amplifiers of redox signaling. β-cells require proper redox signaling already in cell ontogenesis during the development of mature β-cells from their progenitors. We bring details about redox-regulated signaling pathways and transcription factors being essential for proper differentiation and maturation of functional β-cells and their proliferation and insulin expression/maturation. We briefly highlight the targets of redox signaling in the insulin secretory pathway and focus more on possible targets of extracellular redox signaling through secreted thioredoxin1 and thioredoxin reductase1. Tuned redox homeostasis can switch upon chronic pathological insults towards the dysfunction of β-cells and to glucose intolerance. These are characteristics of type 2 diabetes, which is often linked to chronic nutritional overload being nowadays a pandemic feature of lifestyle. Overcharged β-cell metabolism causes pressure on proteostasis in the endoplasmic reticulum, mainly due to increased demand on insulin synthesis, which establishes unfolded protein response and insulin misfolding along with excessive hydrogen peroxide production. This together with redox dysbalance in cytoplasm and mitochondria due to enhanced nutritional pressure impact β-cell redox homeostasis and establish prooxidative metabolism. This can further affect β-cell communication in pancreatic islets through gap junctions. In parallel, peripheral tissues losing insulin sensitivity and overall impairment of glucose tolerance and gut microbiota establish local proinflammatory signaling and later systemic metainflammation, i.e., low chronic inflammation prooxidative properties, which target β-cells leading to their dedifferentiation, dysfunction and eventually cell death.

## 1. Redox Homeostasis in β-Cell Development and Maturation

### 1.1. Sources of Reactive Oxygen Species and Antioxidants in β-Cells

**Figure 1:**
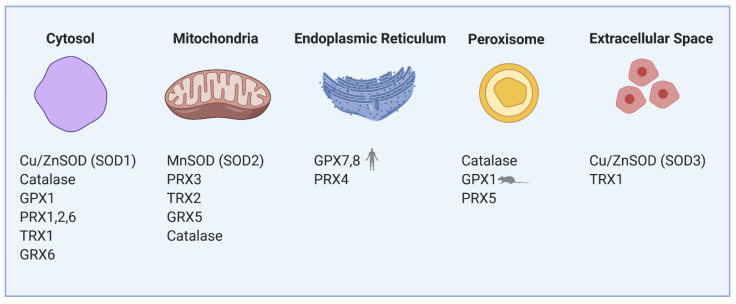
Compartmentalization of redox regulating proteins in β-cells involves mitochondria, cytosol, endoplasmic reticulum (ER), peroxisomes and extracellular space. SOD—superoxide dismutase, GPX—glutathione peroxidase, PRX—peroxiredoxin, TRX—thioredoxin, GRX—glutaredoxin.

Reactive oxygen species (ROS) are normally produced during the metabolism of β-cells and play an important role in cellular signaling. For instance, mitochondrial ROS are obligatory signals of glucose-induced insulin secretion (GSIS) [1,2]. However, excessive levels of ROS in both human and rodent β-cells cause oxidative stress, which is detrimental to the cells. Several such conditions leading to excessive ROS generation in β-cells have been proposed, such as hyperglycemia, hypoxia, hyperlipidemia and endoplasmic reticulum (ER) stress [3]. Pancreatic β-cells both of rodents and humans are reportedly determined to be especially vulnerable to oxidative damage [4] due to the low expression of classical antioxidant enzymes—catalases, glutathione peroxidases (GPX) and superoxide dismutases (SOD) when compared to other cell types [5,6,7]. The main antioxidant system in β-cells consists of peroxiredoxins (PRX), thioredoxins (TRX) and thioredoxin reductase (TRXR). Regeneration of PRX thiol groups is mediated by auxiliary enzymes TRX and glutaredoxins (GRX). The recycling of TRX is mediated by the activity of TRXR, which reduces TRX and allows the cycle to continue. NADPH serves as an electron donor for the reduction of TRXR [8]. GRX is reduced by glutathione, which is then regenerated by glutathione reductase with the concurrent utilization of NADPH [8]. This system was shown to be sufficient to protect β-cells against short-run oxidative burst while hypothetically also providing a signaling role necessary for GSIS in both rodent and human cells [9]. Nevertheless, long-term glucolipotoxic conditions, often caused by overnutrition leading to an increase in oxidative stress, can cause dysfunction of the β-cells and contribute to type 2 diabetes (see Section 3). The redox balance between ROS and the antioxidant system is, therefore, crucial for the proper physiological function of pancreatic β-cells, and thus body glucose homeostasis. Because of the internal redox compartmentalization in β-cells, we have focused on the local sources of ROS and types of antioxidant enzymes in individual cell parts (Figure 1). ROS are primarily produced during mitochondrial oxidative phosphorylation but also originate from other cell compartments and organelles, such as ER, peroxisomes or cytoplasm [10,11]. At a “redox triangle” called redoxosome, which is formed by closely attached membranes of the ER, mitochondria and peroxisomes, redox-regulatory enzymes are thought to assemble. These sense ROS accumulations and redox imbalances, and its enzymes use ROS to transmit intercompartmental signals via chemical modifications of downstream proteins and lipids [12]. Under physiological conditions, ROS production in individual compartments is well controlled by specific resident antioxidant enzymes with small differences between rodents and humans.

#### 1.1.1. Mitochondria

Mitochondrial electron transport chain (ETC) complexes I and III produce superoxide (O_2_^•−^) into the mitochondrial matrix and complex III also into the cytosol (exactly to the intracristal space). Mitochondrial MnSOD (*Cu*/*ZnSOD* isoform in the cytosol) transforms O_2_^•−^ into a more stable molecule H_2_O_2,_ which is then reduced to water by an antioxidant enzyme GPX, mitochondrial PRX3 or catalase [13]. Under physiological conditions in the presence of glucose, mitochondrial production of ROS is not detrimental to the β-cells. GSIS does not increase mitochondrial ROS production in rodent β-cells [2,14,15]. However, elevated H_2_O_2_ derived from superoxide formed by fatty acid oxidation (specifically by electron transfer flavoprotein-ubiquinone oxidoreductase) activates mitochondrial phospholipase iPLA_2_γ. iPLA2γ-cleaved fatty acids are being utilized by UCP2 to induce mild uncoupling and subsequently reduce respiratory chain-mediated superoxide formation. Such an antioxidant feedback mechanism based on redox signaling initiated by fatty acid oxidation in rodent β-cells might suppress further ROS production and oxidative stress. Also, iPLA2γ-cleaved fatty acids were shown to amplify insulin secretion in mice through the GPR40 receptor [16,17]. This way, mitochondria may help the cells to cope with the nutrient overload.

#### 1.1.2. Endoplasmic Reticulum

**Figure 2:**
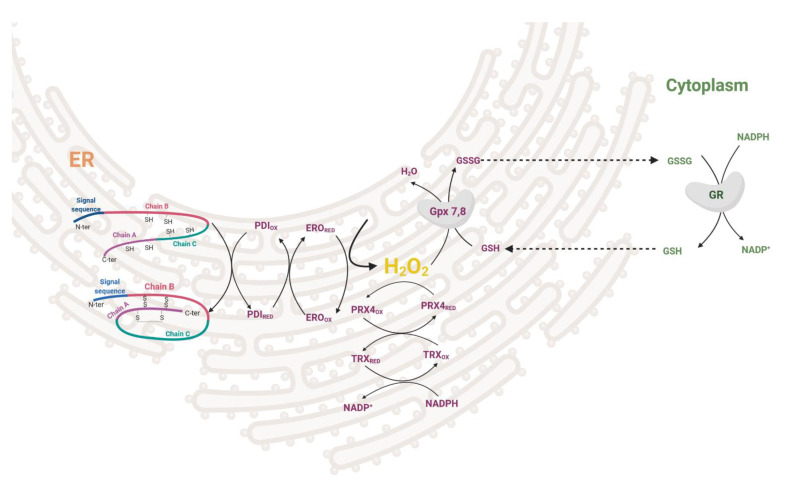
Redox regulation in ER during proinsulin folding. Disulfide bonds are formed by disulfide isomerase (PDI) together with ER oxidoreductin (ERO) oxidoreductases while giving rise to H_2_O_2_. This can be attenuated by PRX4/TRX or GPX7,8/GSH redox couples in conjunction with cytosolic redox status. PDI—disulfide isomerase, ERO—ER oxidoreductin, PRX—peroxiredoxin, TRX—thioredoxin, GPX—glutathione peroxidase, GSH—glutathione.

During the maturation of insulin in the ER, three disulfide bonds are formed by disulfide isomerase (PDI). After the formation of the S–S bond, PDI needs to be regenerated to its oxidized form. This is enabled either by ER oxidoreductin-1 (ERO1), which accepts electrons from PDI and donates them to oxygen resulting in the formation of H_2_O_2_ molecules or by GSSG-driven oxidation of substrate proteins through PDI (Figure 2). The interplay between the two oxidative pathways that produce or consume ER-luminal GSSG maintains ER redox status (manifested in human HEK293 cell line) [18]. H_2_O_2_ is then metabolized by PRX4 in both rodents and humans, together with GPX7/8 in humans [19,20,21,22]. Overexpression of PRX4 in rat INS-1E cells protects against ROS toxicity and increases insulin content by affecting ER protein folding capacity [19]. Redox balance on the ER level affects other membranes of the redoxosome. Long-term overproduction of ROS in ER leads to cell stress, activation of unfolded protein response, β-cell dedifferentiation and subsequently apoptosis (see Section 3.2). The state of protein folding in ER is controlled by chaperones of the Hsp70 and Hsp40 families, chaperone-like proteins, and lectins [23,24]. The key ER members of the Hsp70 family are represented by chaperone immunoglobulin heavy-chain-binding protein, BiP (also called GRP78) [25]. UPR signaling is mediated via three main transmembrane sensors: endoribonuclease inositol-requiring protein 1α (IRE1α), protein kinase RNA-like endoplasmic reticulum kinase (PERK), and activating transcription factor 6 (ATF6) [26]. When misfolded proteins accumulate in the ER, BiP dissociates from the UPR sensors and binds to the exposed hydrophobic domains of the unfolded proteins [27]. This induces oligomerization and auto-transphosphorylation of IRE1α and PERK, with their consequent activation [28].

#### 1.1.3. Peroxisomes

In peroxisomes, ROS are produced mainly during the β-oxidation of long-chain polyunsaturated fatty acids. Because of the weak antioxidant defense of peroxisomes in both rodents and humans, β-cells are unable to endure long-term peroxisomal stress. Catalase expression is rather suppressed to allow specific β-cell function [5,6]. Also, *Prx5* is weakly expressed [20]. Thus, weak detoxifying machinery in peroxisomes leads to uncontrollable ROS production in lipotoxic conditions. However, the peroxisomal loss causes mitochondrial deterioration and cytoplasmic vacuolization in β-cells impairing glucose tolerance [29].

#### 1.1.4. Cytoplasm and Membrane Rafts

In our previous research on mice [2], we have shown that after glucose uptake, the ROS production arises mainly in the cytoplasm, where specific H_2_O_2_ producers are localized: NADPH oxidases (NOX) and xanthine oxidases (XOD) [30,31,32]. These enzymes have an important role in the physiology but also the pathophysiology of pancreatic β-cells. Under physiological conditions, the cytoplasmic antioxidant system is fully sufficient to maintain the redox balance. During GSIS, the levels of NADPH and mitochondrial FADH_2_ are increased, NAD(P)H-dependent ROS production is stimulated, and detoxifying enzymes (NOX, XOD and PRX/TRX/TRXR system, respectively) are activated. The primary cytoplasmic antioxidants are PRX1, PRX2 [33] and, to a lower extent, also Cu/ZnSOD, catalase and GPX1 [5,6,11,20] (Figure 1). Thioredoxin interacting protein (TXNIP) was suggested to increase oxidative stress by binding TRX and inhibiting its reducing activity [34]. Upregulated by glucose, TXNIP regulates β-cell antioxidant defense and redox homeostasis. Carbohydrate response element-binding protein (ChREBP) participates in TXNIP expressional regulation, thus linking redox balance to glucose sensing through the ChREBP–TXNIP–TRX axis both in humans and rodent β-cells [35]. Besides TRX, many other proteins were suggested to be the binding targets to the TXNIP protein; thus, TXNIP may be a scaffold protein important in redox signaling [36]. As discovered on human cancer cell line MCF-7, one of TXNIP’s binding partners is importin-α, which allows the complex to directly enter the nucleus [37]. TXNIP overproduction during long-time hyperglycemia has a deleterious effect on β-cell function also through controlling microRNA (miR) expression. Through upregulation of miR-204, TXNIP downregulates expression of V-maf musculoaponeurotic fibrosarcoma oncogene homolog A (*MafA*), one of the major insulin transcription factors [38]. Induction of another miR-124a expression elevates the production of islet amyloid polypeptide, further aggravating the Langerhans islets pathology [39].

### 1.2. ROS Signaling Pathways and Transcription Factors in β-Cell Physiology

The exact mechanisms by which ROS (mainly H_2_O_2_) serve as signaling molecules and metabolic coupling factors in well-functioning β-cells are still not fully understood. However, there is strong evidence of ROS importance in proper β-cell function [40]. It was found that administration of exogenous H_2_O_2_ and enhancing intracellular H_2_O_2_ levels increases insulin release in rat INS-1 (832/13) cells and both rat and mouse islets [41,42,43]. On the contrary, the application of exogenous antioxidants inhibits GSIS [43]. How could H_2_O_2_ (or other ROS) mediate its signaling effects with the antioxidant system present in the cell? In general, three main mechanisms of H_2_O_2_ function have been proposed. First, there may be strong localized intracellular H_2_O_2_ gradients that enable local H_2_O_2_ signaling despite the mean cytoplasmic concentration being low. Second, PRX may be locally transiently inactivated during signaling, allowing H_2_O_2_ levels to increase. Third, the action of H_2_O_2_ may be mediated by redox relays whereby PRX and/or glutathione peroxidases and catalase to less extent act as initial H_2_O_2_ sensors and then oxidize the end target proteins [44]. Redox sensing and signaling within the cells are mediated via reversible disulfide-dithiol exchange reactions and de-nitrosylation of Cys residues. The process is enzymatically controlled by members of the thioredoxin family comprising over 50 proteins, including TRX, PRX, PDI, glutathione-dependent glutaredoxins (GRX), GPX and glutathione-transferases [45]. The enzymes interact with many distinct proteins affecting cellular functions like gene expression [46], regulation of intracellular H_2_O_2_ levels [33], proliferation [47], apoptosis [48] and modulation of the immune response [49].

#### 1.2.1. Redox Sensitive Signaling Pathways

Several cellular pathways were shown to be affected by redox status in β-cells. Signaling pathway KEAP1–NRF2–ARE, in general, maintains the cellular redox balance and induces adaptive response to oxidative stress [50]. This pathway consists of three main components: Kelch-like ECH-associated protein 1 (KEAP1), nuclear factor erythroid 2-related factor 2 (NRF2), and antioxidant response elements (ARE). Under physiological conditions, KEAP1 is associated with NRF2, and the ubiquitination of NRF2 is stimulated. However, under oxidizing conditions, ROS promote the dissociation of KEAP1 and NRF2, enabling NRF2 to transfer to the nucleus, where it binds to ARE and enhances the expression of detoxification enzymes, such as glutathione synthetase, glutathione reductase, GPX, TRX, TRXR and PRX to prevent oxidative stress [50]. Elevated NRF2 activity has a positive effect on β-cell function during glucolipotoxicity in mice [51]. However, under physiological conditions, insulin release is reduced after activation of NRF2 [51]. Chronic H_2_O_2_ treatment is known to activate KEAP1–NRF2–ARE signaling to promote the production of pro-apoptotic factors and leads to apoptosis of human pancreatic β-cells [52]. ROS were also shown to activate the nuclear factor kappa-light-chain-enhancer of activated B cells (NF-κB) pathway. NF-κB, when activated, translocates to the nucleus and works as a transcription factor that affects many cellular processes. This pathway can influence the redox status by increasing the expression of antioxidant proteins, such as *Cu*/*ZnSOD*, MnSOD, GPX, etc. [50]. However, NF-κB activation in pancreatic β-cells has mostly deleterious effects and contributes to the loss of differentiated β-cell functions and leads to cell death [53,54,55] (more in Section 3). Another major intracellular signal transduction pathway that plays an important role in various cellular processes is the mitogen-activated protein kinase (MAPK) cascades. This includes the extracellular signal-regulated kinase (ERK) pathway, the c-Jun N-terminal kinase (JNK) pathway, the p38 kinase pathway and the big MAP kinase 1 (BMK1/ERK5) pathway. ROS were reported to activate these pathways under physiological and pathophysiological conditions of β-cells [50,56].

#### 1.2.2. Redox Regulation of Transcription Factors Essential for β-Cell Maturation

**Figure 3:**
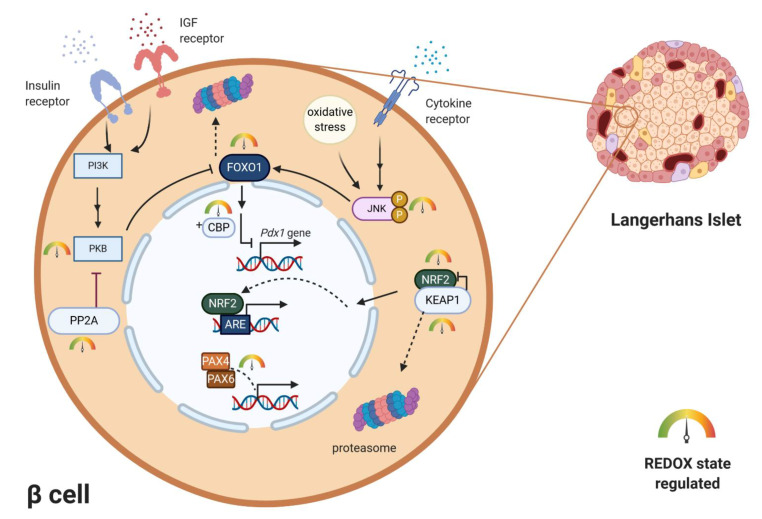
Examples of redox regulation of specific transcription factors. *Pdx1* gene is negatively regulated by redox-sensitive transcription factors FOXO and CBP. Localization and transcription activity of FOXO is negatively regulated by PI3K-PKB/AKT signaling and positively regulated by the JNK signaling pathway. Activated by oxidative stress/cytokine stimuli, JNK enables FOXO nuclear localization. Concurrently redox-sensitive PKB is inhibited by H_2_O_2_-induced intramolecular disulfide bond formation. Redox sensitive NRF2/KEAP1 system regulates expression of key antioxidant enzymes upon ARE region. Under oxidizing conditions, the cytoplasmic complex dissociates, NRF2 enters the nucleus and functions as a transcription factor. Redox-sensitive cysteines in PAX transcription factors directly regulate their DNA-binding ability. ARE—antioxidant response elements, CBP—p300/cAMP-responsive element-binding protein (CREB)-binding protein, FOXO—forkhead box O protein, IGF—insulin-like growth factor, JNK—c-Jun N-terminal kinase, KEAP1—Kelch-like ECH-associated protein 1, NRF2—nuclear factor erythroid 2-related factor 2, PAX4/6—paired-homeobox genes, PDX1—pancreas/duodenum homeobox protein 1, PI3K—phosphoinositide 3-kinase, PP2A—protein phosphatase 2A, PKB—protein kinase B.

For a long time, activation of gene transcription has been considered to be primarily, if not exclusively, regulated by cascades of protein phosphorylation and dephosphorylation [57]. The concept that transcription might be controlled by redox reactions emerged when an important eukaryotic transcription factor–NF-κB–was found to be activated by oxidants and inhibited by antioxidants [57,58,59]. Several theories how oxidants could affect transcription were suggested—oxidative inactivation of phosphatases in signaling cascades, direct oxidation of transcription factors, activation of protein kinases, redox-dependent noncovalent binding of thioredoxin, thiol modifications of proteins that form cytosolic complexes with transcription factors, or heterodimer formation of GPX and PRX-type peroxidases with transcription factors. In the mature pancreas, transcription factors play a role in achieving glucose homeostasis by regulating the expression of key genes, most notably the insulin gene [60]. Mature β-cells of all species express high levels of transcriptional factors. The main ones include Pancreas/duodenum homeobox protein 1 (PDX1), MAFA, homeobox protein NKX6.1 and transcription factor neurogenic differentiation (NEUROD). All of these transcription factors were shown to be downregulated by persistent ROS in rodents and human β-cells [3]. However, these changes occur under pathophysiological conditions of oxidative stress and often lead to cell damage. PDX1 is essential in the development of β-cells and also binds to the regulatory elements and increases insulin gene transcription and several other islet-specific genes like glucose transporter2 (*Glut2*) or *Nkx6.1* [60]. It contributes to β-cell mass expansion and glucose metabolism induced by activation of protein kinase B (PKB/Akt) signaling [61]. The activity of PDX1 was shown to be reduced by oxidative stress [62,63]. Another critically important activator of insulin gene transcription, MAFA, is sensitive to proteasomal degradation under conditions of oxidative stress in rodents [64,65]. The major regulator of MAFA degradation under oxidative stress is p38 MAPK, which directly binds to MAFA and triggers MAFA degradation via the ubiquitin proteasomal pathway. Inhibiting MAFA degradation under oxidative stress in rodents ameliorates β-cell dysfunction [66,67]. Reduction of intranuclear MAFA levels was observed in diabetic *db*/*db* mice. This adverse effect was prevented by β-cell-specific GPX1 overexpression, which induced β-cell antioxidant protection [68]. Both of the above-mentioned transcription factors are regulated by forkhead box O protein 1 (FOXO1) (). FOXO1 belongs to the FOXO family, which has an important role in many cellular processes, including resistance to cellular oxidative stress itself, in cellular metabolism and insulin signaling [69]. FOXO can sense ROS levels by oxidation of its cysteines and then regulates the activity of transcription factors. In humans, ROS induces the formation of cysteine-thiol disulfide-dependent complexes of FOXO and the p300/cAMP-responsive element-binding protein (CREB) binding protein (CBP) acetyltransferase, thus modulating FOXO biological activity [70]. Multiple FOXO connected pathways have been shown to be subject to regulatory cysteine oxidation [71]. The insulin and insulin-like growth factor (IGF) signaling pathways inhibit FOXO activity by activation of phosphoinositide 3-kinase (PI3K) and subsequently PKB [72] (Figure 3). Studies performed mainly on rodents showed that phosphorylation of FOXO by PKB causes FOXO inactivation and its export from the nucleus [69,71]. Under oxidative stress, H_2_O_2_-induced oxidation of PKB leads to the transformation of its Cys297 and Cys311 into intramolecular disulfide bonds [73,74], dephosphorylation of PKB and an increased association with protein phosphatase 2A (PP2A) (Figure 3). The higher affinity of PKB to PP2A leads to faster inactivation of this kinase, which enables nuclear localization of FOXO [71]. Interestingly, activated FOXO1 negatively regulates *Pdx1* expression during oxidative stress in βTC-3 cell culture and mice [75]. It was also discovered that under oxidative burst, FOXO1 can bind to promyelocytic leukemia protein (PML) and NAD-dependent deacetylase sirtuin-1 (SIRT1) forming a single complex with a slightly inverse effect. This complex upregulates *Ins2* gene transcription factors MAFA and NEUROD expression [76]. MAFA has an important role in β-cell development and replication. Shift from MAFB to MAFA expression during early development in β-cells has a significant role in β-cell maturity determination, replication and survival (reported on rodent model) [77]. This way FOXO1 in the short-term protects β-cells against oxidative stress and tries to preserve their proper function [3,76]. More investigations have to be made to clarify this dual effect of FOXO on β-cells. Apoptosis signal-regulating kinase 1 (ASK-1) is also activated in a redox-dependent manner. ASK-1 activates JNK, which leads to phosphorylation of FOXO and prevention of its export from nucleus [71]. Family of paired-homeobox genes (PAX) proteins (paired-homeobox genes) being crucial for islet development also occurs to be sensitive to redox regulations. PAX8 loses its ability to bind DNA molecules after oxidation of its cysteines Cys45 or Cys57. These cysteines are conserved in all Pax members, so the redox regulation occurs also in human and rodent β-cells, where PAX4 and PAX6 are present [78,79,80].

### 1.3. Redox Regulation of β-Cell Differentiation and Proliferation

#### 1.3.1. Redox Regulation of β-Cell Differentiation

During differentiation of β-cells from embryonic stem cells, strictly controlled repression or activation of specific transcription factors and genes that manage the transition occurs. During the embryonic days 8.5–12.5, expression of specific transcription factors is activated: *Sox17, Pdx1, Hb9, Isl1, Hnf1β, Foxa2, Hnf6, Gata4 and Gata6*. Between embryonic days 12.5–16.5, other transcription factors are activated: *Ngn3, NeuroD1, Pax4, Pax6, Hes1, Hnf6, Sox9, Ptf1a, Rfx3, Rfx6, Isl1, Arx, Nkx2.2, Nkx6.1, Glis3, MafA and MafB* [81,82,83,84]. Mutations of these genes and their deficiency during early embryonic days lead to reduced β-cell mass, hyperglycemia and development of diabetes mellitus in rodents [84]. Moreover, new approaches in the treatment of diabetes rely on the conversion of differentiated adult cells or progenitors into β-cells in a process called cellular reprogramming and the promotion of their further proliferation [85]. Identifying and understanding key regulators specific for the development and identity of β-cells is, therefore, necessary. Redox signaling was suggested to be one of these regulators as treatment of pancreatic progenitors by low ROS levels leads to β-cell formation and its viability increase [86,87]. In murine embryos, H_2_O_2_ production positively correlates with β-cell formation. Moreover, NOX4 production of H_2_O_2_ stimulates the differentiation of β-cells progenitors accompanied by positive effects on *Sox9* and *Ngn3* gene expression in vivo and in vitro. Low levels of H_2_O_2_ promote the expression of differentiation markers *Ngn3* and *Nkx6.1* and markers of maturation [88]. On the contrary, reducing H_2_O_2_ concentration leads to a decrease in β-cell development, viability and proliferation [86,89] and overexpression of catalase, specifically in mice mitochondria of β-cells leads to a significant reduction in β-cell proliferation and volume [87]. β-cell differentiation is mediated probably through modulation of the ERK1/2 signaling pathway, which controls proliferation, differentiation and also phosphorylation and protein levels of CREB [89,90]. CREB, together with its coactivator CBP participates in β-cell gene expression and proliferation. In mutated mice and isolated islet cultures, constitutively activated cAMP signaling and enhanced CREB–CBP binding leads to an increase in proliferation and doubling β-cell mass [91]. The human *SOX9* proximal promoter is also regulated by the CREB in other cell types [92]; thus, the positive effect of H_2_O_2_ from NOX4 on *Sox9* and *Ngn3* genes could be connected to the activation of the ERK1/2 signaling. In other cell types, the influence of H_2_O_2_ on the activation of signaling pathways connected to β-cell proliferation was discovered: PI3K/PKB pathway [93] and WNT signaling pathway [94].

#### 1.3.2. Adipokines Stimulate β-Cells Proliferation through Redox Signaling

Hormones leptin and adiponectin are secreted by adipose tissue and play a major role in the homeostasis of the organism and β-cell condition. They control body weight, food intake and overall metabolism [95,96]. Many studies have been performed to elucidate the effects of leptin and adiponectin on β-cells; however, results have been controversial [96]. There is a negative feedback between insulin and leptin secretion. Insulin promotes leptin release, and leptin then inhibits insulin production and secretion [97] by opening the K_ATP_ sensitive channels [98] and inhibiting protein phosphatase 1 [99], important in vesicle docking and exocytosis (well discussed in [100]). The effect of adiponectin on insulin secretion seems to rely on glucose concentration. Studies on rodent and human models showed that adiponectin does not affect basal insulin secretion; however, it stimulates insulin secretion at high glucose [96,101]. Adiponectin improves insulin sensitivity in target tissues due to the suppressive effect on gluconeogenesis and positive influence on glucose uptake and fatty acid oxidation in muscle and liver [96]. However, the exposition of β-cells to elevated levels of these adipokines leads to a significant increase in their proliferation and viability. This is due to the increase in NOX activity and thus an increase in ROS production, which was confirmed by using exogenous antioxidants. The positive effect of adipokines on ROS elevation is accompanied and supported by a decrease in the *Cu*/*ZnSOD* expression and Cu/ZnSOD, GPX and catalase activity in rodents [86].

#### 1.3.3. ROS-Mediated Ca^2+^ Signaling in β-Cell Proliferation

Due to a direct effect of ROS on redox-sensitive Ca^2+^ channels [11,102], ROS-mediated intracellular rise in Ca^2+^ concentration can lead to activation of calcineurin, which potentiates both human and rodent β-cell proliferation [103,104]. Calcineurin phosphorylation of the Transducer of regulated CREB activity-2 (TORC2) leads to its translocation into the nucleus and association with other transcription factors (CREB, cAMP-responsive element modulator (CREM) and cyclic AMP-dependent transcription factor ATF-1 (ATF1)) leading to enhanced expression of cell-cycle-activating genes [103]. At low nutrient levels, when ROS production and Ca^2+^ signaling in β-cells are generally low, AMPK kinase member microtubule affinity regulating kinase 2 (MARK2) is activated. MARK2 has an opposite effect on TORC2 than calcineurin; thus, starvation blocks β-cell proliferation [103].

## 2. Redox Homeostasis in Optimum β-Cell Function

### 2.1. Redox Signaling in Insulin Secretory Pathway

**Figure 4:**
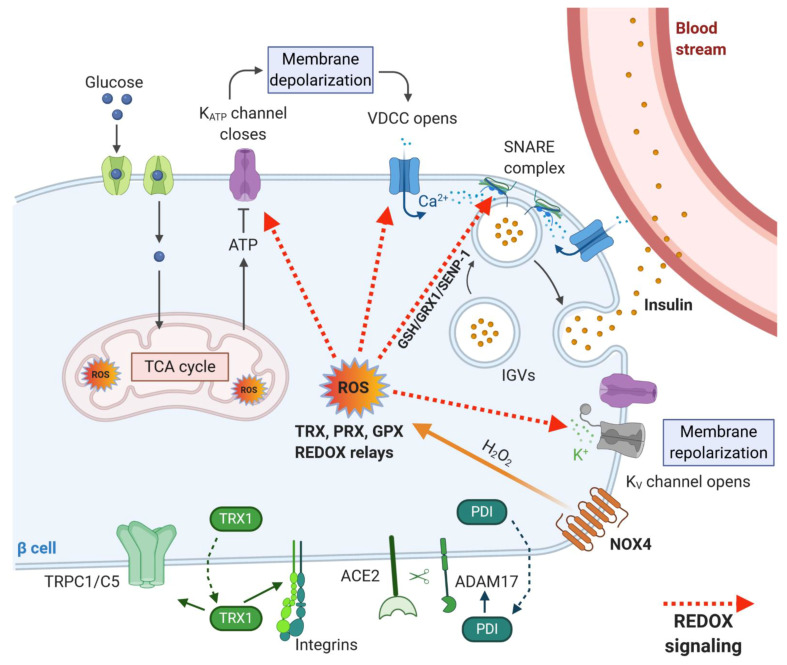
Redox targets within insulin secretory pathway and proteins involved in extracellular redox signaling. Glucose entry through GLUT1 in humans or GLUT2 in rodents causes an increase in ATP/ADP ratio and elevation of ROS production in the cytoplasm (mediated by NOX4). Subsequently, the K_ATP_ channel closes, which causes depolarization of the plasma membrane, the opening of voltage-dependent calcium channels (VDCC) and Ca^2+^ entry into the cells. Ca^2+^ is required for insulin granule vesicles (IGV) trafficking, attachment and fusion with the plasma membrane (mediated by SNARE proteins) and finally, secretion of insulin from the cell. Repolarization of the membrane is mediated via voltage-gated K^+^ channels (K_V_). These repeating cycles lead to a high-frequency of action potential spikes and synchronized oscillations in the cytosolic Ca^2+^. ROS are utilized by redox-regulated proteins TRX, GPX and PRX, which can either neutralize the ROS action in the long-term or transform acute redox signal via redox relays. Targets of redox signaling involve K_ATP_ channels, VDCC, K_V_ channels and proteins involved in granule exocytosis. *TRX1* and PDIs are secreted from β-cells, and they interact with extracellular domains of transmembrane proteins. PDIs regulate the action of sheddase ADAM17, and *TRX1* modifies the activity of integrins and TRPC1/5 channels.

β-cells can adapt the rate of insulin secretion to the plasma concentration of glucose and other nutrients by a unique coupling system between nutrient metabolism and insulin production. The coupling system involves accelerating glycolysis, and mitochondrial metabolism with rapid changes in the oxidation state of several redox couples, e.g., NADH/NAD^+^ and NADPH/NADP^+^ and is also directly linked to ROS production as mentioned above. Upon higher substrate load, the demand for oxygen supply to the cells grows as well. At such a high metabolic activity, the oxygen demand exceeds oxygen supply, resulting in the establishment of intracellular transient hypoxia [105]. Such features have been described, for example, in neurosecretory cells, which require high mitochondrial activity and ATP production to restore resting membrane potential and to maintain intracellular Ca^2+^ levels [106] and also in working skeletal muscle cells, in which increased ATP demand is reflected by mitochondrial biogenesis and elevated oxygen consumption resulting in expression of hypoxia-inducible genes [107]. Hypoxia could serve as another coupling factor via enhanced ROS production for insulin secretion in β-cells. Moreover, the beneficial effect of hypoxia in healthy β-cells could be attributed to the biogenesis of the mitochondrial respiratory chain proteins [107] and effective calcium signaling [106], which are inevitable for the maintenance of mature β-cells. It was also suggested that the basal activity of hypoxia-induced pathways is necessary for normal β-cell function [108]. An interesting study by Olsson and Carlsson [109] has shown that oxygenation may differ widely between individual islets within the pancreas at a given time point. These differences may reflect a mechanism to recruit only a fraction of the available islets into an active (normoxic) β-cell mass. The remaining less well-oxygenated (hypoxic) islets may represent a dormant subpopulation, constituting a functional reserve of endocrine cells. According to this model, the reserve islet pool may be available for recruitment upon reduction of the total islet mass [109]. In any case, the importance of redox signaling in GSIS machinery was also documented by our research group, which showed recently that insulin secretion is inhibited upon deletion of constitutively active NOX4 enzyme in rodent β-cells in vivo. Our results demonstrated that the sole increase in ATP/ADP ratio is not sufficient to stimulate insulin secretion in vitro and in vivo. We thus confirmed that the presence of ROS, namely H_2_O_2_ as a metabolic coupling factor, is essential for insulin secretion [2]. Many targets of redox signaling in the insulin secretory pathway were suggested (Figure 4); however, a more detailed/complex view is still missing. K_ATP_ channel with its essential function in GSIS [110] mediating depolarization of plasma membrane was shown to be inhibited by H_2_O_2_ in smooth muscle cells [111], but no such regulation has been observed in β-cells. Also, another depolarizing transient receptor potential (TRP) channel subfamily M member (TRPM2) was reported to be directly redox activated [112]. The subsequent repolarizing events on the plasma membrane seem to be potentially redox-regulated as H_2_O_2_ could directly or indirectly inhibit repolarizing K^+^-channels, such as K_V_ [113,114,115]. Besides redox targets of signaling pathways mentioned above, downstream aims in the secretory pathway were found to be, for example, redox-sensitive proteins of calcium signaling, e.g., ryanodine receptor 2 (RyR2), sarcoplasmic Ca^2+^ ATPase (SERCA) and inositol triphosphate receptor (IP3R) [11,102]. The glucose-induced changes in the redox environment affect these proteins, leading to increased exocytosis of insulin secretory vesicles. Further, proteins of the secretory machinery were also implicated to be redox-regulated. For example, redox-regulated posttranslational modification of exocytosis-regulating *t*-SNARE proteins has been proposed to result in an increased rate of maturation, or priming, of secretory vesicles in yeasts (for an overview, see [116]). mRNA/protein expression studies and electrophysiological analysis of exocytosis suggested that both the expression level of *Grx1* and *Trx1* in the β-cell as well as the NADPH/NADP^+^ redox status are important factors for the regulation of exocytosis [117]. Redox-dependent regulation was suggested for the NADPH–GSH–GRX1 signaling axis in rodent β-cells [118], and GRX1-GSH was shown to mediate deSUMOylation of sentrin/SUMO-specific protease 1 (SENP1) in humans [119] via modulation of its key thiol groups [120]. SUMOylation/deSUMOylation processes were previously reported to be redox-regulated and were implicated to be involved in response to oxidative stress [121]. Moreover, SUMOylation of insulin secretory pathway proteins had an inhibitory effect on insulin granule exocytosis, and several proteins involved in insulin granule exocytosis were shown to be modified by SUMOylation [121]. However, it is tempting to speculate that the redox signal mediated by the thiol exchange is transformed at the exocytotic site to signal mediated via SUMO posttranslational modification.

### 2.2. Extracellular Redox Signaling in β-Cell Function

It was shown that many oxidoreductases could be secreted from the cells to the extracellular milieu as a response to changes in the cellular environment [122]. The involved secretory pathways differ based on secreted enzyme and cell type. GRX1 is secreted by human T-cells, B-cells and NK-cells of the immune system [123]. Different PDIs are secreted by platelets playing a crucial role in thrombosis [124]. PRXs were detected to be secreted by macrophages and HEK297 cells [125]. TRX1 is secreted by many different cell lines, including human fibroblasts, epithelial cells, cells of the immune system, hepatocytes and cancer cells [126]. Interestingly, current research revealed secretion of cytoplasmic isoforms of TRX1 and TRXR1 by murine and porcine pancreatic β-cells during hypoxia and inflammation [126]. Such phenomenon was originally described in immune cells and presence of TRX1 was detected in blood of patients suffering from rheumatoid arthritis [127] and diabetes [128]. Extracellular TRX1 seems to cause autocrine or paracrine regulation of the mouse β-cells as recombinant TRX1 injected to mice with transplanted pancreatic islets significantly improved cell viability and improved blood glucose control, while overexpression of TRX1 in grafted islet cells did not lead to such effect [129]. In other experiments extracellular TRX1 had beneficial effect on murine and porcine β-cells by preventing apoptosis and preserving insulin secretion [126]. It is also possible that binding of TXNIP to TRX1 could negatively affect its secretion and subsequent extracellular actions. It is highly likely that except from TRX1 β-cells secrete also other oxidoreductases and that all these enzymes contribute to extracellular redox signaling towards other endocrine cells. More experimental evidence in this field will definitely reveal remarkable findings in future.

Until now, there is only a very limited number of transmembrane proteins, which were described as targets of extracellular redox modifications by secreted oxidoreductases. PDIs were pointed out as inactivators of ADAM metallopeptidase domain 17 (ADAM17) shedding activity [130,131,132]. ADAM17 is a disintegrin and metalloproteinase that participates in the cleavage of surface proteins. An increase in ADAM17-mediated shedding activity and decrease in its substrate angiotensin-converting enzyme 2 (ACE2) have been observed with the progression of type 2 diabetes mellitus (T2D) [133], suggesting the importance of this mechanism in the disease. Indeed, restoration of ACE2 improved glycemia in *db*/*db* and Ang-II-infused mice. The beneficial effects of ACE2 could be attributed to reduced oxidative stress and ADAM17 expression in the islets of Langerhans, in addition to the improvement of blood flow to the β-cells [134]. It seems probable that the regulation mediated by the PDIs–ADAM17–ACE2 axis may play an important role in sensing the redox environment in the β-cells and their surrounding, resulting in signaling events leading to maintenance of the proper β-cell functionality.

Another group of surface proteins described as targets of TRX1 and PDIA6 are the integrins [135,136]. Integrins are transmembrane heterodimer receptors for extracellular matrix (ECM) components. They are expressed on all cell types and composed of one *α* and one *β* subunit. Integrins affect a variety of cell processes, including adhesion, migration, differentiation, and cell growth. In mammals, 24 *α* and 9 *β* subunits have been identified, which combine to create 24 unique heterodimers [137]. Both the *α* and *β* subunits are involved in the determination of ligand specificity; however, the *β* subunit facilitates adhesion and activates intracellular second messenger cascades [138]. Most notably, stimulation of the *β*1 integrin leads to activation of focal adhesion kinase (FAK), ERK, PKB, and the steroid receptor coactivator (SRC) family of kinases, all of which are involved in cell survival. Moreover, integrins also mediate crosstalk with growth factor receptors. The signals that are activated by the integrin–FAK–SRC complex can be integrated with signals from growth factor receptor binding to subsequently activate the RAS–MAPK kinase–MAPK pathway, which is canonically associated with cell proliferation and survival [139]. Ligands for integrins include fibronectin, laminin, collagen, fibrinogen, and thrombospondin [137]. Regulation of disulfide formation and cleavage by TRX1 and PDIs could, therefore, affect β-cell growth, proliferation and survival- processes mediated by integrins.

The last group of redox-regulated transmembrane proteins that must not be forgotten are TRP channels. They comprise six divergent subfamilies TRPV, TRPC, TRPM, TRPA1, TRPP and TRPML, with diverse biophysical properties and functions. The TRP channels have a common structure of homo- or heterotetramers with six transmembrane spanning regions and intracellular N- and C-termini. TRP channels are sensitive to changes in the microenvironment, including heat, osmotic pressure, mechanical force and changes in the redox environment of the cells. They were found to be sensitive to redox modifications (for an overview, see [112]). It has been reported that the extracellular reduced thioredoxin activates homomeric TRPC5 and heteromeric TRPC5–TRPC1 channels by breaking a disulfide bridge in the predicted extracellular loop next to the ion-selectivity filter of TRPC5 [140]. It has been shown that TRPC5 and TRPC1 are expressed in secretory fibroblast-like synoviocytes from patients with rheumatoid arthritis, whose extracellular concentration of thioredoxin is high. The endogenous TRPC5–TRPC1 channels of the cells are activated by reduced thioredoxin, and the blockade of the channels enhances the secretory activity and prevents the suppression of secretion by thioredoxin. As patients with diabetes also have high levels of extracellular TRX1, one can predict that a similar mechanism also exists in pancreatic β-cells. It was also documented that activation of multiple subtypes of TRP channels results in enhanced insulin production by β-cells, and redox signal causes activation of the channels [141].

## 3. Dysbalanced Redox Homeostasis Contributing to β-Cell Dysfunction under Metabolic Stress

### 3.1. Redox-Linked Transition to Dysfunctional β-Cell in T2D Pathology

Chronic overnutrition and a sedentary lifestyle are currently increasing the incidence of T2D. As previously stated, glucose metabolism transiently increases oxidative status in β-cells required for efficient insulin secretion; however, long-term substrate pressure modulates redox homeostasis towards persistent prooxidative as β-cells show low detoxifying capacity. Actually, classical antioxidant machinery (catalase, glutathione peroxidases) is compensated by peroxiredoxins, thioredoxins, haem oxygenases, metallothioneins, etc., also having a signaling role [142]. To emphasize the role of redox status in the physiology and pathology of β-cells, we searched the GEO Datasets database for potential redox genes that are dysregulated in patients with type 2 diabetes compared to normal healthy individuals. The gene expression datasets of GSE20966, GSE25724, GSE26168, GSE38642 were incorporated. These datasets include expression profiling from array data from type 2 diabetic and non-diabetic isolated human (both males and females) islets and β-cell-enriched tissue obtained by laser capture microdissection. We revealed nine “redox” genes that were either up-or downregulated in the patients suffering from T2D (Table 1), further validating the importance of the cell redox balance in maintaining the precise physiological function.

Overnutrition leading to chronically overcharged metabolism impacts the insulin biosynthetic pathway, where ER overload and activation of the unfolded protein response pathway (UPR) are affected. Metabolism of β-cells adjusts to higher supply and fuel availability, increasing operating flux capacity and changing characterization of mitochondrial function and overall metabolism. Redox systems then adapt and respond to these changes by altered intracellular redox communication among pyridine nucleotides, ROS and thiols [143]. Rapid/chronic responses to nutrient intake can be observed further through the blood redox metabolome, thus exceeding intracellular compartments [143]. In parallel, a nutritional surplus is stored in peripheral tissues, mainly adipose tissue, which, when its capacity is exceeded, starts to be stored in other peripheral tissues, such as liver, muscle, etc., soon establishing insulin resistance. Precipitous increase of fat mass is accompanied by altered secretion of adipokines contributing to the reinforcement of systemic inflammation. The pro-inflammatory environment shows prooxidative behavior, further contributing to β-cell dysfunction. Information within cells is shared via the common metabolic cofactors, which are used by many pathways and enzymes [144]. Systemic inflammation/metainflammation, along with local proinflammatory activity in the pancreas, white adipose tissue, etc., is the key pathological determinant of T2D etiology. Here, otherwise, positive immunomodulators, such as macrophages, production and activity of IL1β cytokine and gut microbiota composition switch their activity under chronic nutrient conditions to proinflammatory, which further harmfully targets β-cells. The physiological activity of β-cells then starts to lose its identity, its function while compensating for insufficient insulin secretion by increasing β-cell mass. Later, β-cell metabolism trying to keep glucose homeostasis switches from hyperfunction to hypofunction. They continue to dedifferentiate into less mature β-cells, general endocrine cells or bi/multi-hormonal endocrine cells, thus reducing their β-cell mass where in late-stage of T2D or in parallel β-cells are significantly eliminated by cell death. Altered redox environment may affect not only individual β-cells, but also communication channels between individual β and other endocrine cells in pancreatic islets leading to desynchronization of β-cell signaling in islets and suppression of effective insulin secretion.

### 3.2. Affected Redox Homeostasis Impacts Insulin Biosynthesis in β-Cells

Extensive insulin biosynthesis requires robust ER, which produces more than 1 million molecules of insulin per minute in a single β-cell [145]. Upon overnutrition, ER is overloaded, and induced stress activates UPR. UPR then restores folding homeostasis/proteostasis in β-cell experiencing manageable, often temporary, levels of ER stress. Once the threshold is exceeded, e.g., under chronic nutritional conditions, UPR triggers cell degeneration, alters β-cell differentiation and induces apoptosis through inositol-requiring enzyme 1α (IRE1α) kinase/endoribonuclease (RNase) accompanied by ROS production [146,147]. Affected maturation of insulin can reflect the failure of redox homeostasis within the cytoplasm of β-cell being set towards prooxidation. A recent in vitro study showed that mitochondrial antioxidant MitoQ efficiently reduced ER stress (documented by GRP78 and P-eIF2α markers) and proinflammatory NFκB-p65 signaling under hyperglycemic conditions [148].-folding of proinsulin in ER involves the establishment of three disulfide bonds (B7-A7, B19-A20, A6-A11) within the proinsulin chain. Substantially oxidizing equivalents from cytosol are transferred to the ER lumen mediated through ERO1 to PDI and proinsulin, as stated above. Affected cysteine pairing or altered redox ER status can thus cause misfolding and accumulation of toxic aggregation of proinsulin in ER. Proinsulin folding is facilitated by 15–20 PDIs in conjunction with ERO1α/β. The later one then regenerates PDIs for subsequent rounds of disulfide bond generation (more in Section 1.1.2). Disulfide formation in the ER is dependent on the cytosolic NADPH–thioredoxin redox system, which is tightly reflected by changes in glucose metabolism. Furthermore, extensive oxidative folding machinery produces a large amount of ROS and depletes the GSH pool, thus further contributing to redox disbalance towards prooxidation [149]. This is evident from using Akita mouse islets, where expression of the mutant form of proinsulin, which is prone to misfolding, further increase oxidative stress in β-cells [150]. ER stress is also able to induce the activity of TXNIP through the PERK and IRE1 pathway, leading to NLR family pyrin domain containing 3 (NLRP3) protein inflammasome formation and induction of cytokine secretion, thus promoting inflammation [151]. Such a vicious cycle amplifies the disruption of ER function, thus proper insulin maturation and redox homeostasis. It is known that early T2D and prediabetes are accompanied by an accumulation of misfolded proinsulin. Thus, imbalanced redox homeostasis, which otherwise relies on the continuous supply of reducing equivalent, is crucial for the proper function of the β-cell, especially insulin secretion [152]. Another evidence supporting the role of redox status in ER biosynthetic machinery showed that chronic overnutrition and obesity increasing blood pro-atherogenic oxidized low-density lipoproteins (LDL) cholesterol, then initiates oxidatively induced ER stress. H_2_O_2_ mimicked oxidized LDL induction of *Chop/CHOP* and *p58IPK/P58IPK* (ER stress markers) and was blocked by co-treatment with *N*-acetyl cysteine (NAC) [153]. Thus, redox homeostasis in β-cells has an impact on insulin biosynthesis, and its disbalance upon chronic nutrient overload accelerates β-cell dysfunction. As a part of the adaptive phase of UPR, crosstalk between ER and mitochondria by calcium fluxes through mitochondria-associated membranes (MAM) sites has been shown to modulate energy consumption, increasing ATP synthesis in mitochondria [154]. Thus, permanent ER stress also affects calcium homeostasis, which can further have an effect on mitochondrial function and derived redox status eventually alters insulin secretion machinery. Moreover, sustained calcium uptake to mitochondria can sensitize β-cells to cell death.

### 3.3. Adipose Tissue Hypertrophy-Induced β-Cell Dysfunction

**Figure 5:**
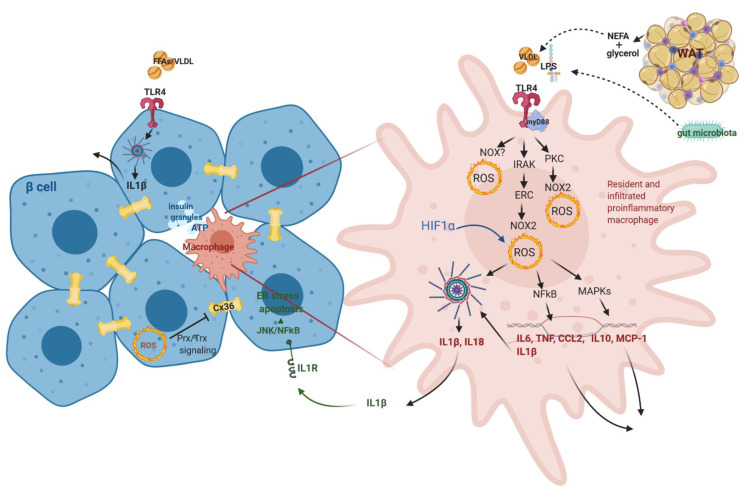
Activation of proinflammatory macrophages involves prooxidative signaling. Altered insulin secretion from β-cells signals to activation and differentiation of resident and infiltrating macrophages in islets, the latter one being also activated through TLR4 by lipoproteins from WAT and microbiota-derived metabolites. Activation induces prooxidative metabolism required for inflammasome formation and production of proinflammatory cytokines/chemokines, which target β-cells towards dysfunction. Upon chronic nutritional overload, also β-cells can form inflammasome and secrete IL1β. Gap junctions (composed of CX36) between β-cells of the pancreatic islet are suggested to reflect β-cell redox status and thus regulate islet insulin secretion. TLR4—Toll-like receptor 4, WAT—white adipose tissue, IL1β—interleukin 1 β, CX36—connexin 36.

Chronic nutritional overload causes adipocyte hypertrophy and hyperplasia, mainly in white adipose tissue (WAT). Hypertrophic adipocytes have a blunted response to insulin and progressively become more lipolytic, thereby releasing an excess of non-esterified fatty acids (NEFA) to circulation [155]. These compounds together with lipoproteins then reach β-cells and induce their inflammation mostly through toll-like receptor 4 (TLR4) on islet macrophages and β-cells further involving myD88-NF-κB/MAPK signaling pathway along with induction of prooxidative signaling inducing expression and release of TNFα, IL6, IL1β, etc. [156] (Figure 5). The establishment of prooxidative status can be suggested to be regulated through the TLR4-PKC-NOX pathway or TLR4-IRAK-ERC-NOX2 or TLR4-NOX directly [157,158,159] (Figure 5). Moreover, it was suggested that oxidative stress established in adipose tissue in the early to middle stages of obesity is induced mainly by NOX recruiting macrophages to inflamed tissue, while the later stages involve mitochondrial ROS production maintaining the proinflammatory environment and contributing to insulin resistance [160,161]. The inflammatory cellular state and redox homeostasis is also guarded by antioxidant induction through NRF2/KEAP1/ARE pathway [162]. The rapid increase of WAT mass establishes hypoxia, further inducing the production of cytokines, chemokines and other proinflammatory mediators, which are secreted to circulation, maintain a proinflammatory systemic metabolic state characterized by increased C-reactive protein and aberrant cytokine generation. For example, adipose tissue macrophages are the main source of IL6, with an estimated contribution of 15–35% of the total circulating IL6 [163]. Hypertrophic WAT, its chronic inflammation, and ER stress further accelerate the recruitment of immune cells and the production of proinflammatory metabolites through 12/15-lipoxygenases (more in [164]). Altered adipokines production upon chronic overnutrition modulates the levels of leptin and adiponectin, which have an impact on insulin sensitivity, content, secretion and vascular function (for an overview, see [165]). When the storage capacity of WAT exceeds the threshold, ectopic fat deposition takes place. Those can be the liver, pancreas, muscles, etc. Under these conditions, the pancreas was shown to increase triglyceride content in T2D pathology, where lipotoxic pancreatic lipids impair insulin secretion and induce insulin resistance [166].

### 3.4. Impaired Redox Homeostasis of the Immune System Leading to Β-Cell Dysfunction

#### 3.4.1. Macrophage Role in Langerhans Islets- from Guarding Β-Cell Activity to Its Dysfunction

Resident macrophages (CD11b^+^Ly6C^−^ or F4/80^high^CD11c^low^) in pancreatic islets guard the activity of β-cells [167]. Their depletion in healthy islets from lean chow-fed mice was shown to decrease GSIS [167]. The authors monitor β-cell function through the content of secreted insulin granules containing insulin, ATP, C-peptide and serotonin, being a potent stimulator of immune cells and proliferation and/or regeneration of β-cells [168,169,170]. In the case of acute injury of β-cells or under higher insulin demand, they slowly proliferate and release connective tissue growth factor as a chemoattractant to recruit further macrophages, which then facilitate the recovery/increase of β-cell mass [171]. These are bone marrow-derived macrophages recruited to the site of injury. They, together with resident macrophages, provide signals (e.g., PDGF) driving β-cell hyperplasia to overcome insulin insufficiency adapting to early weight gain and the development of insulin resistance [167]. The main communication molecule was suggested to be ATP as ATP released from over-functional β-cells induces anti-inflammatory cytokine IL10 and matrix metallopeptidase 9 (MMP-9) production from resident/perivascular macrophages in islets through purinergic signaling (Figure 5). Matrix metalloproteinase MMP-9 activity is important for β-cell function, islet vascularization, reduction of cellular inflammation and degradation of islet amyloid. Weitz et al. showed that in obese and diabetic states, purinergic receptor expression in macrophages, thus the response to ATP, is downregulated [168]. Under the pathological condition of T2D, the number of macrophages is increasing where mainly circulating macrophages (CD11b^+^Ly6C^+^ recruited from circulating monocytes) are infiltrating into islets, but also F4/80^low^CD11c^high^ proliferating macrophages in situ [156,167,172,173]. CD3^+^ cells (T lymphocytes) were not found to be significantly increased. Thus obesity-associated islet inflammation is dominated by macrophages, with negligible involvement of adaptive immune cells [167,174]. Their depletion improves GSIS in HFD fed obese mice [156,167,173]. They can be activated through TLR4 by nutritional triglyceride-rich lipids (palmitate, VLDL, etc.), which upregulates intracellular levels of ceramides and potentiate the pro-inflammatory response [175]. Inflammatory macrophages are characterized by significant prooxidative behavior and metabolic adjustment (increased production of citrate, succinate, etc.), leading to the production of many cytokines (TNFα, CCL2, IL6, etc.) where IL1β deserves special interest in respect to β-cell dysfunction since β-cells show very high expression of its receptor in the membrane [176] (Figure 5). Production of mature IL1β involves activation of inflammasome through the AMPK–autophagy–mitochondrial/cytosolic ROS signaling axis [177]. β-cells can also produce IL1β, probably initiating the inflammatory process (see in Section 3.4.2); however, macrophages are seemingly the major source of islet IL1β production in obesity [172,178]. Intracellular impairment of β-cell function by inflammation involves JNK and NF-κB signaling pathways. NF-κB activation in β-cell can, for example, reduce expression of calcium pumps of ER, leading to ER stress and decreased GSIS [179]. JNK activation can lead to suppression of insulin receptor substrate/IRS-PI3K-PKB signaling, which further increases nuclear localization of FOXO1, which decreases binding to PDX1 and thus reduces insulin production and secretion [180,181]. IL1β contributes to insulin resistance through TNFα-dependent and -independent mechanisms [177].

#### 3.4.2. IL1β Dual Role in β-Cell Function/Dysfunction

IL1β signaling is associated with proinflammation in most tissues; however, β-cells show dual effects being integral for cell physiology and inflammation. IL1β is crucial for β-cell activity, as knockout for the receptor for IL1β in vivo cause β-cell dedifferentiation [182], while chronic accumulation or elevated production of IL1β is rather proinflammatory and detrimental to β-cell GSIS. High IL1β concentrations suppress the expression of genes associated with fully differentiated β-cell phenotype and increase the number of bi-hormonal cells in islets [183]. IL1β β-cell dysfunction depends on ER stress (PERK activation), calcium release and JNK signaling [184]. This, together with reduced expression of IL1 receptor antagonist, accompanies T2D pathology [176]. The physiological effect of IL1β on β-cells is evident. The number of peritoneal macrophages increases postprandially, and secreted IL1β induces insulin secretion. This effect depends on the bacterial product (translocation of an ingested or intestinal bacterial product rather than by microbiota itself) and, together with enhanced glucose metabolism and derived prooxidative intracellular status (through TXNIP or PI3K), enable IL1β maturation. IL1β then activates PKB/PI(3)K pathway, glucose uptake via glucose transporter 1 (GLUT1) and insulin secretion [185,186]. Insulin also increases glucose uptake by macrophages, making loopback [187]. However, under chronic overnutrition, long-term upregulation of IL1β leads to elevated insulin levels, which keeps macrophages in the inflammatory state. Early-stage increased insulin plasma concentration might also contribute to low chronic grade inflammation. Insulin preferentially acts on inflammatory macrophages having more expressed insulin receptors, enhanced phosphorylation of PKB and glycolytic activity [187]. In parallel, chronic glucose/nutrient overload of β-cells affecting redox homeostasis leads to activation of inflammasome assembly and further IL1β processing towards mature protein, which is then released (Figure 5). This can recruit further macrophages from circulation and propagate inflammation [167,188]. Inflammasome thus serves as a critical sensor of nutrient overload. The deletion of the NLRP3 inflammasome improves β-cell physiology and viability during oxidative stress and hypoxia that may be associated with anti-inflammatory effects, such as attenuated macrophage islet infiltration [189], however, show probably deleterious effect in physiological conditions.

### 3.5. Gut Microbiota Supervision of the Immune System

The intestinal microbiota is recognized as a strong immunomodulator. Under the development of the metabolic disease, obesity or T2D microbial dysbiosis is established, associated with the expansion of underrepresented microorganisms (usually opportunistic pathogens) and lower phylogenetic alpha diversity. Such bacteria express proinflammatory behavior (e.g., gram-negative *E. coli*) at the expense of anti-inflammatory, e.g., *Faecalibacterium prausnitzii, Roseburia,* etc. [190,191,192]. In obese individuals, proinflammatory bacteria can extract more energy from the diet compared to healthy lean controls [193]. Reduced anti-inflammatory bacteria are linked to disturbed short-chain fatty acid (SCFA) production (such as propionate, butyrate, acetate as the results of microbial degradation of fibers) having an otherwise beneficial effect on host metabolism, especially insulin sensitivity through regulating mitochondrial metabolism of peripheral tissues and thus keeping redox homeostasis [194,195]. The effect of SCFA, which enters the cells through FFA2/GPR43 and FFA3/GPR41 receptors, on β-cells is rather controversial [196]. Nevertheless, SCFA can mimic histone deacetylase (HDAC) inhibitors in β-cells, which promotes their proliferation, differentiation and function [197]. Proinflammatory microbes are then able to get through an intestinal wall as its permeability is increasing based on evidence of suppressed expression of tight junction genes in the intestine under HFD [198]. Proinflammatory bacteria show moreover increased adherence to the epithelium, thus enabling local inflammation [199]. Microbiota regulates metabolic inflammation directly through TLR receptors (mainly TLR4/2) present on cells of the immune system (Figure 5). TLR induced NFκB expression along with induced ROS production activates inflammasome, which induces metainflammation.

### 3.6. β-Cell Dedifferentiation as the Adaptation to Metabolic and Redox Changes

There is still a dispute whether and at which stage of diabetes pathogenesis insulin insufficiency is the result of the compromised β-cell function (i.e., the amount of released insulin by individual β-cell) and reduced β-cell mass (i.e., the number of β-cell per individual pancreas) and/or combination of both. A recent study on human pancreas tissue slices of control, prediabetic and T2D individuals by Cohrs. et al. proved evidence that the functional deficit of persistent β-cells is more dominant and an early feature of T2D pathogenesis [200]. They showed that early stages of T2D pathogenesis at which subjects exhibited impaired glucose tolerance and a loss of first-phase insulin release but are not insulin resistant and are not yet diabetic exhibit significant functional deterioration and exhaustion, while their β-cell volume is maintained at this stage of disease progression. β-cells within islets were shown not to be uniformly based on their functional activities. Such heterogeneity may be relevant for T2D pathogenesis [201]. Altered redox status was found to contribute to β-cell dedifferentiation upon chronic nutrient overload, mainly through alteration in insulin gene expression [202] through major transcriptional factors PDX1 and MAFA. Transgenic expression of GPX1 restored MAFA expression in *db*/*db* mice [68,203] while ebselen (GPX mimetic) established the nuclear expression of PDX1, MAFA in diabetic ZDF rats [204]. Redox-regulated nuclear localization and DNA-binding activity of PDX1 involves JNK signaling and FOXO1 [180,205]. FOXO1 seems to play complex adaptive/deleterious function under stress conditions [83]. Under oxidative stress induced by chronic nutrient overload, it translocates to the nucleus and promotes the expression of *MafA* and *NeuroD1* expression [76], while its somatic deletion in β-cells induces ER stress and dedifferentiation with upregulation of progenitor markers [206]. In severely hyperglycemic *db*/*db* mice and insulin-resistant GIRKO mice, FOXO1 is downregulated, and this further affects the expression of MAFA and NKX6.1 [206,207,208]. On the other hand, FOXO1 can stimulate antioxidant protection in the various cellular compartment by expressional induction of antioxidant genes (more in [209]). Thus, FOXO1 is the regulator of cellular redox status and its posttranslational modifications; localization reflects intracellular redox status where detailed induction and signaling in β-cells require further studies [209]. Various subpopulations of β-cells exist differing in responsiveness to glucose, in diverse expression profiles associated with β-cell maturation, glucose metabolism, insulin secretion and pathology of T2D [210]. These subpopulations exist in adult islets and their distribution alters in the process of T2D development. In T2D and long exposure of β-cells to hyperglycemia and metabolic stress, cells tend to differentiate into immature, developmental expression program, which is characterized by low insulin content, poor insulin secretion, gene markers indicating a potential for proliferation [211]. Dedifferentiation is connected to the appearance of forbidden genes, where expressional induction of some of them, such as lactate dehydrogenase (LDH-A) was initiated by oxidative status, either hypoxia-inducible factor (HIF-1) signaling or others [212,213]. Long-term exposure to high glucose due to peripheral glucose intolerance and increased insulin demand induces hypoxia and hypoxia-induced pathways both in cell lines and isolated islets [214]. An increase in hypoxia-related genes, including HIF-1α, vascular endothelial growth factor A (VEGF-A), PAI-1 and LDH-A, was detected in islets of prediabetic Zucker diabetic fatty rats [215], indicating that these pathways are activated during the development of T2D. At normoxia, HIF-1α protein is constantly hydroxylated by prolyl hydroxylases on conserved proline residues [216] and subsequently recognized and degraded by ubiquitination [216]. However, at hypoxia, HIF-1α is no more degraded and dimerizes with the HIF-1β subunit to create a functional dimer, which is transported to the nucleus to execute its transcriptional functions [217]. HIF-1 transcriptional activity regulates a plethora of genes connected with angiogenesis, cell proliferation/survival, and glucose/iron metabolism [218]. One of the main cellular metabolism changes mediated by HIFs is a switch from aerobic to anaerobic energy production. An increase in expression of GLUT1, several glycolytic enzymes, LDH-A and pyruvate dehydrogenase kinase (PDK1) and a parallel decrease in mitochondrial oxygen consumption strengthens the ATP production through the glycolytic pathway [219]. HIF-1α was shown in a recent study to mediate expression of β-cell disallowed genes like *Ldha/LDH-A*, *Glut1/GLUT1*, a marker of dedifferentiation aldehyde dehydrogenase 1 family, member A3 (*Aldh1a3*/*ALDH1A3*/ALDH1A3) and endocrine progenitor cell marker NGN3 and to suppress the expression of β-cell functional genes like *Glut2/GLUT2*, *Neurod1/NEUROD1*, *Mafa/MAFA*, *Nkx6.1* and *Pdx1* suggesting that hypoxia-induced pathways are involved in β-cell dedifferentiation [220]. Long-term hypoxia in β-cells results in cell-death, typically by necrosis [221] and also induction of apoptosis-related pathways upon hypoxia exposure has been described in β-cells [222,223]. Recently, a new group of transcription factors called inhibitor of differentiation protein (IDs) was found to be a novel class of oxidative stress-responsive genes in β-cells enhancing antioxidant pathway through NFE2L2-small MAFs pathway [224]. Dedifferentiation and possible transdifferentiation of endocrine cell types were described as the reason for the presence of polyhormonal cells in islets of T2D subjects [225]. Thus, various subgroups of β-cells undergo gradual cell fate transition while adapting the metabolic gene expression profile to function given a particular metabolic status in response to the glycemic and metabolic situation. They adapt to discrete roles within functional islet syncytium [201]. For example, increased substrate pressure and metabolic stress on β-cells caused by overnutrition and low-energy expenditure might stimulate some β-cell subpopulations towards proliferation through FOXO1 signaling in order to adjust to enhanced insulin demand [226]. When the pressure on β-cells becomes chronic, other factors like inflammation are induced, there is no way back, and exhaustion of β-cells leads to their functional deterioration and death evidenced in late-stages of T2D pathology. β-cells seem to be metabolically flexible, and thus, the identification and characterization of β-cell subpopulations in islets represents a powerful tool for targeting treatment of T2D into earlier phases of its development, which might then enhance the success of T2D therapy.

### 3.7. β-Cell Communication in Islets under Redox Regulation

**Figure 6:**
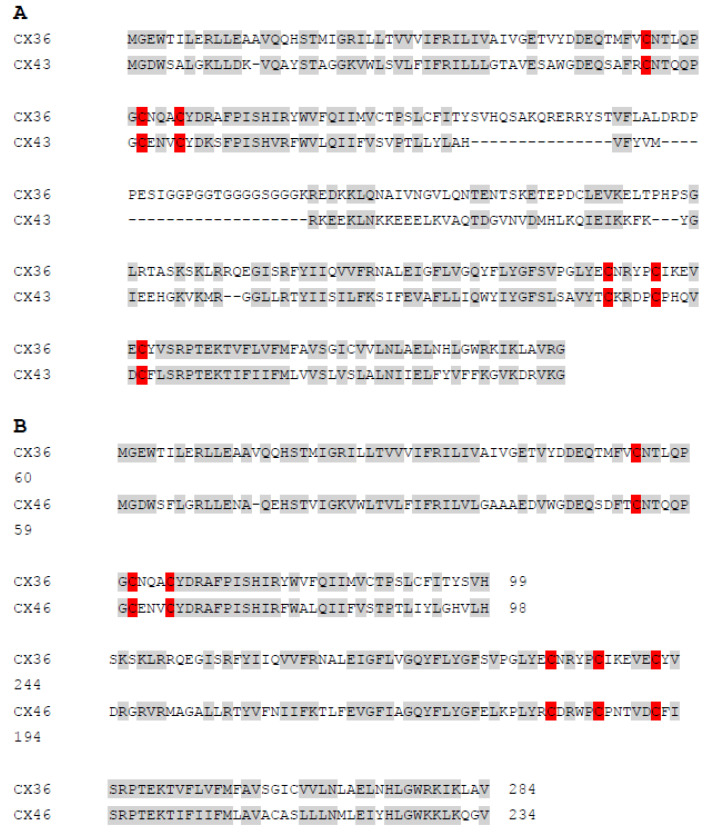
Human CX36 amino-acid sequence alignment with CX43 (**A**) and CX46 (**B**); identical and similar amino acids are depicted in grey, Cys residues-potential targets to redox modifications are highlighted in red; the primary amino-acid sequences were aligned using protein blast tool available at https://blast.ncbi.nlm.nih.gov/Blast.cgi, accessed on 23 January 2021.

Each β-cell responds to glucose or other stimuli autonomously; however, the interaction among groups of β-cells was shown to be tightly controlled and contributes to efficient insulin secretion upon stimuli induction. Involvement of interaction with other endocrine cells will further orchestrate tight control of insulin secretion. Although the spatiotemporal distribution of β-cells with other endocrine cells in pancreatic islets differs between rodents and humans, β-cells always assemble to groups of various numbers, and their proper communication is a prerequisite for insulin release [227]. β-cells exhibit heterogeneous electrical activity, which needs to be synchronized with the related intracellular calcium oscillations all over the islet to achieve pulsatile insulin release [228]. Gap junctions between adjacent cells play a fundamental role. Recent studies applying optogenetic and photopharmacological methods enriched the knowledge about the dynamics of the β-cell network in humans and rodents [229,230]. They uncovered the existence of few subpopulations of β-cells with reduced β-cell identity (lower levels of PDX1, INS1) but more sensitive to glucose (increased expression of glucokinase) called hubs, which drive the response of the remaining β-cells called followers through cellular coupling [231,232]. This was found to be conserved across species (including mice, humans and fish) [201]. However, consensus needs to be reached about the role of hubs as pacemakers for the islet activity. Those hub β-cells showing enhanced sensitivity to glucose might be the reason why these cells are more susceptible to diabetic insults and their loss of function might be critical for T2D development. In any case, functional gap junction coupling is essential for this process. β-cells are linked through gap junctions, which are composed of connexin36 (CX36) [233] (Figure 5). It provides electrical coupling between β-cells and thus promotes the regulation of electrical activity and insulin secretion. CX36 gap junctions enable Ca^2+^ oscillations to be coordinated across the islets. Its deletion significantly affects the synchronization of calcium oscillations, and CX36-deficient mice show disrupted first-phase and pulsatile second phase of insulin release [234,235]. CX36-dependent signaling may be thus disrupted under prediabetic conditions. In general, connexin-based channels comprise hemichannels and gap junction channels. The opening of gap junction channels permits the diffusional exchange of ions and molecules between the cytoplasm and contacting cells. Recently, it was hypothesized that connexin composed gap junction and hemichannels properties might be also redox-regulated through extracellular and intracellular cysteines based on the evidence that reduced intracellular free radical production along with high reduced glutathione decrease opening probability of hemichannel [236]. Although this was the case of CX43 and CX46 of blood vessel wall and oocytes, respectively, with possible implication for other cell types., i.e., astrocytes, retina and kidney, we might suggest that possible redox regulation might be valid also in the case of β-cell gap junctions. This might further be involved in insulin secretion efficiency response as suggested above. This assumption is based on amino-acid sequence alignments of CX36/CX43 and CX36/CX46, which shows high similarity in cysteine positions (in red) and the sequence especially between CX36 and CX43 (Figure 6). The sequence of CX36 shares 56% sequence similarity with CX43. The sequence of CX36 shares up to 74% of sequence similarity with CX46, however, the central part of CX36 ( position of residues 109–138, 145–154 and 167–175) is missing in CX46 and CX46 contains a much longer C-terminus, which decrease the sequence similarity of CX36/CX46 to 33%.

**Table 1:** Dysregulated genes of redox-regulating proteins in type 2 diabetes mellitus (T2D) patients.

Gene Symbol	Protein Name	Regulation	Dataset	Reference
*TXN1*	Thioredoxin 1	Up	GSE38642	[237,238,239,240]
*TXNR1*	Thioredoxin reductase 1	Up	GSE26168	[241]
*TXNIP*	Thioredoxin-interacting protein	Down	GSE26168	[241]
*PRX2*	Peroxiredoxin 2	Up	GSE25724	[242]
*PRX4*	Peroxiredoxin 4	Down	GSE25724	[242]
*PRX5*	Peroxiredoxin 5	Up	GSE26168	[241]
*PRX6*	Peroxiredoxin 6	Up	GSE38642	[237,238,239,240]
*GPX1*	Glutathione peroxidase 1	Up	GSE26168	[241]
*SOD2*	Superoxide dismutase 2, mitochondrial	Up	GSE26168	[241]

## 4. Conclusions

Redox homeostasis and signaling is essential in the physiology and pathophysiology of β-cells. β-cell redox homeostasis is the equilibrium of a highly compartmentalized, however interconnected, local redox environment. While imbalanced, T2D is quickly developing, as apparent from expressional dysregulation of redox regulating genes in human patients with T2D. Localized redox milieu involves mainly ER required especially for proper insulin folding and calcium handling, mitochondria required preferentially for bioenergetic supply, peroxisomes being important mainly upon conditions of increased lipids in circulation and cytoplasm connecting all compartments. Redox status impacts three major signaling pathways in β-cells. The KEAP1–NRF2–ARE pathway provides major antioxidant protection. NFκB also participates in antioxidant balance but is active mainly upon deleterious insults. MAPK cascades (ERK, JNK, p38, MAPK1, etc.) signal under physiological and also pathophysiological conditions. Downstream targets of signaling pathways are often transcription factors represented by MAFA, PDX1, NKX6.1, NEUROD, PAX, etc. and other proteins (e.g., FOXO1, PPAs), which have the essential role in insulin expression, β-cell differentiation into the mature functional cell and its proliferation. Redox signaling also targets many components of insulin secretory machinery, such as ion channels, calcium homeostasis and vesicle exocytosis, thus being essential for the proper function of β-cells. Intercommunication of β-cells through gap junctions in pancreatic islets strongly reflects redox homeostasis inside β-cells. This is under the supervision of extracellular redox signaling, which affects the activity of integrins and metalloproteinases, being essential for β-cell differentiation, proliferation and sufficient blood supply. Transmembrane TRP channels also serve as signal transducers of the extracellular redox environment towards β-cells. Thus, redox signaling is essential already for β-cells ontogenesis into mature β-cell, for its main function of insulin secretion upon any insult and communication between β-cell syncytium, perhaps also with other endocrine and exocrine cells in pancreatic islets. The above-mentioned physiological signaling utilizes transient alternation in redox status, briefly disturbing redox homeostasis. When an insult altering redox homeostasis toward prooxidative status exceeds the capacity of antioxidant defense in β-cells and becomes permanent, signaling switches to pathological. This can happen by chronic overnutrition when high supply and fuel availability permanently increase metabolic flux establishing prooxidative status. ER stress and activation of UPR then set β-cells towards metabolic stress, inflammasome formation, inflammatory metabolism, impaired insulin secretion, establishing a vicious cycle of oxidative stress. Chronic overnutrition also affects peripheral tissues, first white adipose tissue responding by altered secretion of adipokines, hypoxia, activation of inflammatory macrophages through TLR4, further supporting ectopic lipid storage in liver and muscles and increasing the release of lipids to circulation. This, together with activation of proinflammatory macrophages by gut microbiota, establishes low chronic inflammation, metainflammation, targeting β-cells towards dedifferentiation, pathological signaling and eventually cell death, the markers of T2D.
